# Management in the care of people with HIV in primary health care in times of the new coronavirus

**DOI:** 10.11606/s1518-8787.2022056003876

**Published:** 2022-03-23

**Authors:** Ianka Cristina Celuppi, Betina Hörner Schlindwein Meirelles, Gabriela Marcellino de Melo Lanzoni, Daniela Savi Geremia, Fernanda Karla Metelski

**Affiliations:** I Universidade Federal de Santa Catarina Centro de Ciências da Saúde Programa de Pós-Graduação em Enfermagem Florianópolis SC Brasil Universidade Federal de Santa Catarina. Centro de Ciências da Saúde. Programa de Pós-Graduação em Enfermagem. Florianópolis, SC, Brasil; II Universidade Federal da Fronteira Sul Faculdade de Enfermagem Chapecó SC Brasil Universidade Federal da Fronteira Sul. Faculdade de Enfermagem. Chapecó, SC, Brasil; III Universidade do Estado de Santa Catarina Centro de Educação Superior do Oeste Departamento de Enfermagem Chapecó SC Brasil Universidade do Estado de Santa Catarina. Centro de Educação Superior do Oeste. Departamento de Enfermagem. Chapecó, SC, Brasil

**Keywords:** HIV, HIV Long-Term Survivors, Continuity of Patient Care, COVID-19, Qualitative Research

## Abstract

**OBJECTIVE:**

To understand management practices in the care of people living with the human immunodeficiency virus (HIV) in primary health care in a Brazilian capital, in times of the new coronavirus (covid-19) pandemic.

**METHOD:**

Qualitative research, anchored in the methodological-analytical framework of the grounded theory, constructivist aspect. Data were collected by using intensive online interviews with nurses from health centers and managers of the municipal health department. Data collection and analysis occurred concomitantly in two phases of analysis: initial and focused coding.

**RESULTS:**

They point to the development of best care practices, with emphasis on initiatives for coordination of care, decentralization of clinical management for primary health care services, establishment of protocols and flows, agreement of intersectoral partnerships, use of groups and social networks, use of tools such as teleconsultation and health surveillance spreadsheet and formation of support networks.

**CONCLUSION:**

The Brazilian capital restructured its network of health services with the implementation of clinical and management protocols, seeking to maintain care for people living with HIV. We highlighted the incorporation of non-face-to-face care technologies and the facilitation of routines, as strategies for expanding access.

## INTRODUCTION

The pandemic of the new coronavirus disease (covid-19), caused by the SARS-CoV-2 virus, has caused significant morbidity and mortality worldwide^1,2^. This scenario has fostered discussions about the forms of organization and adaptation of health services in the face of new demands that emerged with the pandemic, seeking to develop tools and strategies to comply with the guidelines and principles of the Brazilian Unified Health System (SUS). Primary health care (PHC) is the priority gateway into the SUS, to order and coordinate the health care network and, therefore, plays a fundamental role in coping with the covid-19 pandemic^3^.

Some axes of PHC action in this context stand out, such as health surveillance in the territories, care for users with suspected or confirmed cases of covid-19 without severity, social support to vulnerable groups and continuity of care directed to priority groups for this level of health care. Thus, the Brazilian model centered on the family health strategy with a territorial focus already has positive impacts on the health of the population and becomes promising as a strategy for community approach, necessary to cope with any pandemic^4^.

The covid-19 pandemic and the actions taken in response to it are expected to have far-reaching consequences in other diseases^5^. The impacts on the care of other ailments are directly related to interruptions in the usual activities and services to meet the demand for covid-19^6,7^.

Studies show that comorbidities associated with chronic conditions are determining factors for covid-19 mortality, which warns about the need for greater care and caution regarding people living with HIV (PLHIV)^8,9^, who, have a greater need for social distancing due to their immunodeficiency status. In places with high HIV loads, care interruptions during the covid-19 pandemic are estimated to cause an increase in HIV deaths of up to 10%^7^.

This scenario emphasizes the importance of attention to the multimorbidity of PLHIV and ensuring the provision of antiretroviral treatment (ART), without detriment to compliance and to clinical follow-up^9^. However, although international institutions and government agencies have already expressed concern about the HIV epidemic over the years, the covid-19 pandemic presents numerous barriers and challenges to the continuity of care^10^.

Considering that, we elected the following question as the guide of the study: How are management practices carried out in the care of PLHIV in times of covid-19 pandemic? This study proposes to understand the covid-19 pandemic, an emerging and impactful phenomenon in world public health, focusing on the best practices of health care of PLHIV, a population considered a priority by health policies. Best practices are those presenting the best results, being useful tools for the dissemination of innovations in care^11^.

Thus, the study aimed to understand the management practices in the care of PLHIV in PHC in a Brazilian capital in times of covid-19 pandemic.

## METHODS

This is exploratory research of qualitative nature, anchored in the methodological-analytical framework of the grounded theory, in the constructivist aspect, idealized by Kathy Charmaz^12^.

The study was conducted in the municipality of Florianópolis, considered the capital with the highest performance in the national program to improve access and quality of primary care, higher percentage of family health strategy coverage and shared and decentralized care offer in PHC to PVHIV, a national reference in the management and provision of PHC services^13^.

Data collection occurred from July to September 2020, initially interviewing nursing professionals from four PHC centers and subsequently managers in the sectors of specialized care, clinic management, care integration, and epidemiological surveillance of the municipal health department. As recommended by the grounded theory, we opted for the initial selection of nurses due to the different competencies assumed by these professionals in health care, considered a leadership for the management of the work of the multidisciplinary team.

The participants who composed the initial sampling were intentionally selected, considering the inclusion criteria of being a care nurse, coordinator, or resident in PHC and having experience in the management of PLHIV care for at least six months at the date of data collection.

The invitation to join the study was made via e-mail and/or by telephone. The informed consent form was sent virtually to the participants and authorized by using Google Forms^Ⓡ^ , a copy was filed with the researcher.

The intensive interviews took place in non-face-to-face format due to the covid-19 pandemic, by video call using the Google Meet communication system^Ⓡ^ . The first sample group had as its initial question: Tell me about care practices directed at PLHIV in times of covid-19 pandemic. This group consisted of 12 nurses.

From the analysis of the interviews of the first sample group, we hypothesized that the practices of care for PLHIV during the covid-19 pandemic were related to the decentralization of clinical management to PHC, the restructuring of services, and the establishment of protocols and scientific evidence guides.

Therefore, the need to constitute the second sample group with managers of the municipal health department from Florianópolis was identified. The interviews aimed to explore aspects of the establishment of clinical management protocols directed at HIV, and mechanisms for the adequacy of health services for the covid-19 pandemic scenario based on the question: Tell me about managing care for people living with HIV/SIDA in times of pandemic. Thus, the inclusion criterion for this second group was working in management positions in the Florianópolis municipal health department for more than six months at the date of data collection.

Professionals who were away from work during the data collection period, regardless of the reason were excluded from both sample groups. At the end of the interviews of the second sample group, data saturation was achieved with five managers and information to support the investigated phenomenon, totaling 17 participants.

The data were collected by the researchers by interviews recorded in audio, which were transcribed into Word^Ⓡ^ and organized for analysis in the NVIVO10^Ⓡ^ software. The average duration of each interview was 29 minutes. To ensure the anonymity of the participants, the speeches were identified by codes. To obtain the data to ensure the quality and reliability of the study, the principles of the consolidated criteria for reporting qualitative research were followed.

The concomitant analysis of the data, according to the guidelines of the constructivist grounded theory, was performed in two phases. In the first, the initial codification was performed, in which the incidents were coded to understand the information from the participants’ meanings and experiences, constituting the first dimensions of the analyzed experience. In the second phase, focused coding occurred, in which the codes of greater expressiveness were grouped, to form abstract categories to synthesize a given data fragment^12^. Memorandums and diagrams were elaborated to assist the analytical development of the data, leading to the understanding of the phenomenon.

This study respected the ethical precepts of research with human beings, according to Resolution No. 466/2012 and its complementary, including the new procedures for conducting research in a virtual environment, according to Circular Office No. 02/2021.

## RESULTS

The Box shows the categories and subcategories that emerged during the analysis process and that, interrelated, support the phenomenon of study.

**Box:** Representation of the phenomenon, category, and subcategories of the study.

Unveiling the best management practices in the care of people living with HIV related to decentralized, shared, and evidence-based care	
Category	Subcategories
Developing better practices for PLHIV care in the face of the covid-19 pandemic	Prioritizing the care of people living with HIV.
	Facilitating scan routines (CD4, viral load) and antiretroviral treatment.
	Sharing the care of people living with HIV among the health team.
	Encouraging social distancing for immunodepressed people in times of pandemic.
	Strengthening the bond with people living with HIV via groups and social networks.
	Agreeing on intersectoral care strategies/initiatives.
	Accompanying people living with HIV by the health surveillance worksheet.
	Identifying decreased screening of new cases in pandemic times.
	Learning to use tools for non-face-to-face clinical management.
	Establishing eligibility criteria for face-to-face consultation and teleconsultation.
	Facilitating access to PHC services with teleconsultation and hosting via digital platforms.
	Using a protocol to guide clinical management in teleconsultation.

*Unveiling management best practices in the care of people living with HIV.*

The prioritization and concern with the provision of care and monitoring of PLHIV stood out.

*Priority service! We have an internal communication* […] *which accounts for priority care for patients with chronic diseases, infectious diseases, and urgent demands*. (G1E03)

The services also adopted the practice of sending requests for exams and prescriptions via mobile, which facilitates access and continuity of care in a scenario of social distancing.

*We’re doing all recipe renewals by teleconsultation on WhatsApp.* (G1E03)

*One thing we’ve done is renew recipes in an easier way. So, the patient doesn’t need an appointment for every renewal. We try to make the recipe for longer if the patient is stable. Sometimes we see that it is time already to make the viral load or CD4 and we give them the test request beforehand* […] *the consultation will be later, when he has the test result.* (G1E04)

With the decentralization of care to PLHIV for PHC, care was consequently shared among the health team, with division of responsibilities and attributions, including in times of pandemic.

*From the moment the patient contacted the team, we keep giving this follow-up, this care, which is usually monthly.* (G1E01)

*This management of care is done shared with the team, we doctors and nurses, we work a lot together. We say we have a shoulder-to-shoulder job.* (G1E04)

The professionals also emphasized the importance of social distancing for immunosuppressed patients, such as PLHIV.

*What’s changed is the entry into the healthcare center. We have a fully organized work process so that people come as little as possible in the healthcare center.* (G1E03)

*The healthcare center became a hostile environment for them* [the PLHIV]. *So, they didn’t want to come, and that was good because we didn’t want them to come either.* (G1E04)

Initiatives related to the expansion of access and the creation of bonds with PLHIV also stood out. The participants point out the different approaches to communication, among which are the groups and social networks.

*Even on Instagram there are PrEP and PEP groups, and they are always sharing information with patients from our network.* (G1E02)

*There is a manual for using social networks with patients and we received the cell phone for each team.* (G1E03)

Thinking about the continuity of care and treatment of PLHIV, a support network was established with non-governmental organizations (NGOs) so that people who could not retrieve the ART at the distribution center could receive them at home or at the nearest health center. The telephone number of four centers for testing and rapid response was disclosed so that users could communicate their difficulties regarding treatment and request help from this support network.

*Throughout the network we have the articulation with GAPA*^a^ *that receives the medicines for people who can’t go get them because of displacement and financial issues.* (G2G02)

*One of the things we did was helping the ART retrieval, which became impossible for some people without public transportation. So, I chose three NGO leaders, held a meeting with them and said, “guys you’re going to have to help list the names and we help with distribution.”* (G2G01)

*We made a lot of divulgation through the local health council, community leaders, residents’ association.* (G1E04)

The use of management tools, such as surveillance spreadsheets, facilitated the monitoring of PLHIV. This instrument stood out by enabling the visualization of all PLHIV in the territory, and their situation of treatment, medication, examinations, consultations, among others.

*We are keeping an eye on the surveillance worksheet because that’s how we will follow-up these patients.* (G1E04)

*The monitoring worksheet has the name, date of birth, date of diagnosis, date of when they started using antiretrovirals, date of last CD4 and result, date of last viral load and result.* (G1E10)

The professionals identified that prioritizing face-to-face care for the management of respiratory symptoms led to a decrease in the screening of new HIV cases, which results from the decrease in routine consultations for the population without ailments, which were disregarded due to the scarce human resources.

*What’s been happening is that our screenings have been slowing down right now. So, I have a patient who has no symptoms and who comes to me for checkup: in case he has no risk situation, I’m telling him to wait a little longer. I have analyzed that our HIV diagnoses in the pandemic have been quite few. If we don’t do serology, we’re going to stop making a diagnosis and leave people with HIV without knowing they have it.* (G1E04)

Furthermore, new ways of accessing the population were incorporated, such as receiving of demands via WhatsApp^Ⓡ^, email, telephone, and video call. Thus, the professionals had to grasp the use and functionality of these technologies and adapt to the new model of care imposed by the pandemic.

*It’s a challenge doing and having people qualified to do that* [teleconsultation]*, because it’s not just having people, sometimes I have a person, but they can’t write well, they have a hard time understanding.* (G1E03)

Thus, teleconsultation was instituted for routine care without severity and that did not require an initial physical examination. In situations that required face-to-face follow-up, users were instructed to attend the health center for scheduled care.

*We make teleconsultation via WhatsApp, both by video, audio, message, you know? In each case we evaluate the best way. And that makes things a lot easier because people have to avoid leaving home. Sometimes, it’s about specific questions of patients that we already follow-up on. So, we can give this support via teleconsultation.* (G1E01)

*If necessary, we weigh risk-benefit and make a face-to-face assessment of the patient.* (G1E07)

The various technologies incorporated in the health work process improved contact with PLHIV to expand their access to services, which previously occurred in the face-to-face modality. The use of digital means for teleconsultation is a promising tool for health services.

*I think these new technologies come to aggregate and facilitate access, this often facilitates and improves the bond in some cases.* (G1E08)

*We have offered teleconsultation to maintain follow-up in the context of the pandemic. So, teleconsultation is a tool that came to add up at this moment when we are instructing people to stay at home.* (G1E04)

A protocol was elaborated authorizing and regulating teleconsultation to collaborate in the care of users during the covid-19 pandemic.

*They created the protocol that tells us what ethical procedures we should perform, how to make a standard evolution, all the guidelines. So, this protocol came to support us because, at first, we knew nothing about it and did not know the over the phone consultation would be.* (G1E05)

We can understand that PHC services were reconfigured in the face of the pandemic scenario, to transform the workflow, clinical, and managerial practices and incorporate new technologies in the care process, ensuring continuity of care to priority populations for the PHC. The decentralization of care added to the greater autonomy of professionals, represent a potential for the development of better health practices.

The Figure shows the meanings attributed to the best management practices in the care of PLHIV, illustrating the initiatives instituted in the study scenario.

**Figure:**
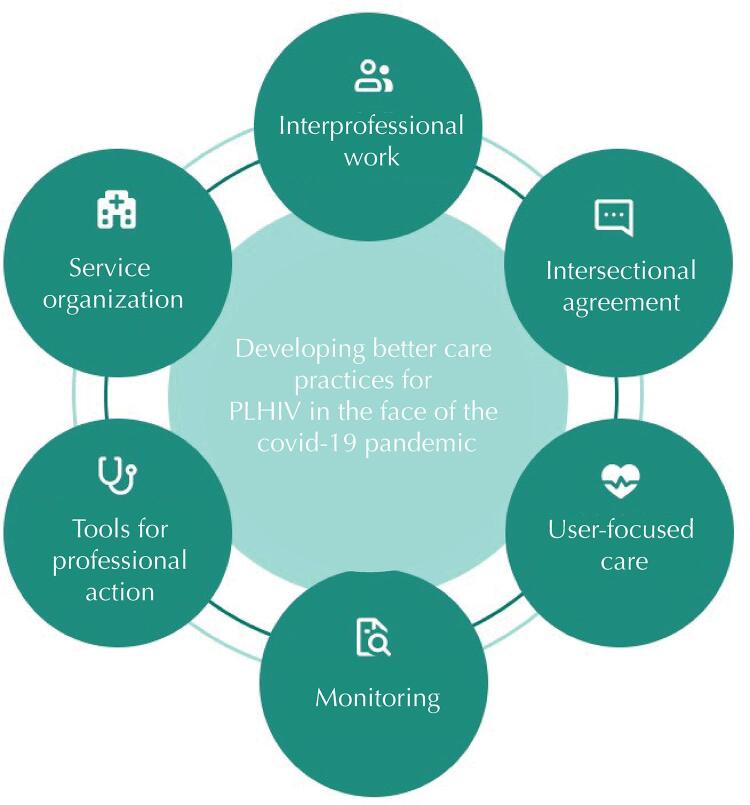
Developing better care practices for PLHIV in the face of the covid-19 pandemic

## DISCUSSION

We understand that the best practices of care for PLHIV identified in this study constitute “innovations in care” and are associated with strategies and tools instituted in PHC in the municipality of Florianópolis, with emphasis on establishing mechanisms of communication and access to services and protocols and guidelines for clinical management, forming intersectoral partnerships, and monitoring and managing remote care. These initiatives promote the coordination of care to PLHIV and reinforce important discussions about the attributes of PHC as the guide of health policy in Brazil.

Reorganizing of PHC services in times of covid-19 pandemic, seeking to simultaneously meet the demand of respiratory symptomatic patients and of regular actions that were already monitored at this level of health care, is imperative to the sustainability of the SUS care model^14,15^. Thus, care flows have been implemented for the management of these different audiences, with a distinction between mild, severe, and non-symptomatic respiratory conditions^4,5^.

All team professionals, especially community health agents, who have a greater approach to the community, should encourage social distancing mobilizing local leaders for the wide dissemination of information and taking measures necessary to the context^4^. Therefore, the vital role of community and intersectoral work in the logistical and operational support of care to PLHIV in times of pandemic is evidenced.

In the scenario of our study, the community and NGO partnerships, such as GAPA, aimed at the transport and distribution of ART, which resulted in access to treatment for countless people who found themselves unable to retrieve it themselves. Studies indicate positive results of the work developed by non-governmental organizations aimed at the prevention, treatment, and monitoring of PLHIV, highlighting improvements in harm reduction, access to condoms and ART, linkage to care and access to health services^16,17^. Articulating solutions in the face of adverse scenarios requires coordinated action in the territory with local leaders, equipment, and institutions, highlighting community engagement and intersectoral actions as important strategies to cope with the covid-19 syndemic^18^.

The concept of syndemic consists of the interaction between two or more epidemics, which produce an increase in the burden of diseases of the population^19^. In other words, the covid-19 pandemic joins the HIV epidemic and creates another health problem for PLHIV, such as decreased access to health services. In this case, with encouraged social distancing and decreased flow of people in health services, the strategy is the management of non-face-to-face care, which can be done with spreadsheets containing clinical data and shared among the health team, and the use of digital communication tools.

The use of management instruments is opportune for maintaining adherence to treatment, identifying gaps and monitoring viral load, CD4, and CD8 tests. However, we highlight some difficulties faced for non-face-to-face clinical monitoring of PLHIV, such as the out-of-date of information, lack of human and material resources, little communication among team professionals, divergent opinions regarding the importance of clinical follow-up, and resistance to changes in work processes^20^.

The covid-19 pandemic has led to the emergence of non-face-to-face health care technologies, such as teleconsultation, which enables the provision of care by using information and communication technologies in the health area^21^. Implementing the teleconsultation in PHC in Florianópolis allowed ensuring access to users who needed health services. Thus, it can be considered a crucial tool of care organization for coping with the pandemic.

We emphasize that teleconsultation should be established in PHC based on protocols that regulate the workflow and establish guidelines^23^, which requires telephony and internet resources, and qualification of professionals and users to take advantage of this new possibility of care^22^.

The use of applications such as WhatsApp^Ⓡ^ and phone calls ensure the offer of actions in a safe way^4^ favoring the continuity of care. The PHC services under study applied these technologies in different situations of PLHIV care, such as to respond to ART related demands, request for routine tests, and receive clinical complaints.

Based on the results of our study, we observe that care management and clinical management, made possible by flows, protocols, and integrated health policy, are fundamental for developing better practices. The restructuring of the health care network and the autonomy, competence and skills of professionals and managers in this scenario of action can effectively intervene in factors that determine the management of health conditions in times of covid-19.

The need for efforts and initiatives to build a strong, vigilant, capillarized PHC, adapted to the pandemic context and faithful to its principles is evidenced. The emerging health crisis with the covid-19 pandemic is sanitary, political, economic, and social, and requires the transformation of the ways of doing health^4^, without forgetting the continuity of the monitoring of priority groups.

Our study analyzed the meanings of the best management practices in care attributed by nurses of health centers and municipal managers, thus presenting limitations regarding the lack of exploration of the perspective of PLHIV on the same phenomenon. Another limitation pertains to the online interviews since we believe that face-to-face interviews could result in greater interaction between interviewer and participant, favoring the deepening of the phenomenon.

Our study contributes to scientific knowledge about the management of care in coping with the covid-19 pandemic and maintenance of PLHIV care, especially regarding the tools, strategies, and technologies used to disseminate the initiatives implemented in the health network studied, which have the potential for replication in other scenarios in which primary health care practices occur.

## CONCLUSION

This study comprised the management practices in the care of PLHIV in the face of the covid-19 pandemic in the PHC in a Brazilian capital, which restructured its network of health services and implemented changes in clinical and management practices to meet the new demands resulting from the covid-19 pandemic and, at the same time, maintain care to PLHIV with quality and effectiveness. The incorporation of technologies and tools for the management of non-face-to-face care as a strategy to expand access and the guarantee of equity and integrality to the care of users in a scenario in which social distancing is reinforced stood out as the best practices.

We highlight the implementation of protocols and flows to guide access to health services to systematize new ways of providing care in times of pandemic, with tools of a digital and technological nature. In this regard, WhatsApp^Ⓡ^ has gained prominence as a tool for receiving demands, doing teleconsultations, requesting exams, renewing recipes, and scheduling of face-to-face activities when necessary.

To that we add the importance of facilitating the routines of performing follow-up exams and retrieving ART. However, the access restrictions resulting from the covid-19 pandemic for routine follow-up of users without aggravated or priority conditions also evidently culminated in the decrease in screening of new HIV cases and control of other chronic conditions.

## Footnotes

a Acronym for AIDS Prevention Support Group, in Portuguese *Grupo de Apoio à Prevenção da Aids*.
