# Supplementary material for: Management in the care of people with HIV in primary health care in times of the new coronavirus
Source: Rev Saude Publica. 2022 Mar 23;56:13. doi: 10.11606/s1518-8787.2022056003876 (PMC8963745; doi:10.11606/s1518-8787.2022056003876)
Supplement: Translation

# Translation: Gestão no cuidado às pessoas com HIV na Atenção Primária à Saúde em tempos do novo coronavírus

**Authors:** Ianka Cristina Celuppi, Betina Hörner Schlindwein Meirelles, Gabriela Marcellino de Melo Lanzoni, Daniela Savi Geremia, Fernanda Karla Metelski

**Affiliations:** I Brasil Universidade Federal de Santa Catarina. Centro de Ciências da Saúde. Programa de Pós-Graduação em Enfermagem. Florianópolis, SC, Brasil; II Brasil Universidade Federal da Fronteira Sul. Faculdade de Enfermagem. Chapecó, SC, Brasil; III Brasil Universidade do Estado de Santa Catarina. Centro de Educação Superior do Oeste. Departamento de Enfermagem. Chapecó, SC, Brasil

**Keywords:** HIV, Sobreviventes de Longo Prazo ao HIV, Continuidade da Assistência ao Paciente, COVID-19, Pesquisa Qualitativa

## Abstract

**OBJETIVO:**

Compreender as práticas de gestão no cuidado às pessoas que vivem com o vírus da imunodeficiência humana (HIV) na Atenção Primária à Saúde de uma capital brasileira, em tempos de pandemia do novo coronavírus (covid-19).

**MÉTODO:**

Trata-se de uma pesquisa qualitativa, ancorada no referencial metodológico-analítico da teoria fundamentada nos dados, vertente construtivista. Os dados foram coletados por entrevistas intensivas online, com enfermeiros de centros de saúde e gestores da secretaria municipal. A coleta e análise dos dados ocorreram de maneira concomitante, em duas fases de análise: a codificação inicial e focalizada.

**RESULTADOS:**

Apontam para o desenvolvimento das melhores práticas de cuidado, com destaque para iniciativas de coordenação do cuidado, descentralização do manejo clínico para os serviços de APS, instituição de protocolos e fluxos, pactuação de parcerias intersetoriais, utilização de grupos e redes sociais, uso de ferramentas como a teleconsulta e planilha de vigilância em saúde e formação de redes de apoio.

**CONCLUSÃO:**

A capital brasileira reestruturou sua rede de serviços de saúde com a implementação de protocolos clínicos e gerenciais, buscando manter a continuidade do cuidado às pessoas que vivem com o HIV. Destacou-se a incorporação de tecnologias de cuidado não presencial e a facilitação de rotinas, como estratégias para ampliação do acesso.

## INTRODUÇÃO

A pandemia da doença do novo coronavírus (covid-19), causada pelo vírus SARS-CoV-2, tem ocasionado morbidade e mortalidade significativas em todo o mundo^1,2^. Diante desse cenário, tem-se fomentado discussões sobre as formas de organização e adaptação dos serviços de saúde frente às novas demandas que emergiram com a pandemia, buscando desenvolver ferramentas e estratégias para cumprir com as diretrizes e princípios do Sistema Único de Saúde (SUS). A atenção primária à saúde (APS) é a porta de entrada prioritária para o SUS, de modo a ordenar e coordenar a rede de atenção à saúde (RAS) e, por isso, desempenha papel fundamental no enfrentamento à pandemia de covid-19^3^.

Destacam-se alguns eixos de atuação da APS nesse contexto, como a vigilância em saúde nos territórios, atenção aos usuários suspeitos ou casos confirmados de covid-19 sem gravidade, suporte social a grupos vulneráveis e continuidade do cuidado direcionado a grupos de prioridade para esse nível de atenção à saúde. Desse modo, o modelo brasileiro centrado na estratégia de saúde da família (ESF) com enfoque territorial, já apresenta impactos positivos na saúde da população e torna-se promissor enquanto estratégia para abordagem comunitária, necessária ao enfrentamento de qualquer pandemia^4^.

Acredita-se que a pandemia de covid-19 e as ações tomadas em resposta, terão consequências de longo alcance em outras doenças^5^. Os impactos no cuidado a outros agravos estão diretamente relacionados às interrupções nas atividades e serviços usuais para o atendimento da demanda de covid-19^6,7^.

Estudos evidenciam que as comorbidades associadas às condições crônicas são fatores determinantes para a mortalidade por covid-19, o que alerta sobre a necessidade de maior cuidado e precaução com relação às pessoas que vivem com HIV (PVHIV)^8,9^, que têm maior necessidade de distanciamento social devido sua condição de imunodeficiência. Estima-se que em locais com altas cargas de HIV, as interrupções do cuidado durante a pandemia de covid-19 possam causar um aumento de óbitos por HIV de até 10%^7^.

Tal cenário enfatiza a importância da atenção à multimorbidade das PVHIV, bem como garantir o fornecimento de tratamento antirretroviral (TARV), sem prejuízos à adesão e ao acompanhamento clínico^9^. No entanto, embora instituições internacionais e órgãos governamentais já venham demonstrando preocupação com a epidemia de HIV ao longo dos anos, a pandemia de covid-19 apresenta inúmeras barreiras e desafios à continuidade do cuidado^10^.

Pensando nisso, elegeu-se a seguinte questão como norteadora do estudo: Como são realizadas as práticas de gestão no cuidado às PVHIV em tempos de pandemia de covid-19? Este estudo propõe compreender um fenômeno emergente e impactante na saúde pública mundial, a pandemia de covid-19, com foco nas melhores práticas de cuidado à saúde das PVHIV, uma população considerada prioritária pelas políticas de saúde. Entende-se por melhores práticas aquelas que apresentam os melhores resultados, sendo ferramentas úteis para a disseminação de inovações no cuidado^11^.

Assim, o estudo teve como objetivo compreender as práticas de gestão no cuidado às PVHIV na APS de uma capital brasileira em tempos de pandemia de covid-19.

## MÉTODOS

Trata-se de uma pesquisa exploratória de natureza qualitativa, ancorada no referencial metodológico-analítico da Teoria Fundamentada nos Dados (TFD), na vertente construtivista, idealizada por Kathy Charmaz^12^.

O estudo foi realizado em Florianópolis, considerada a capital com maior desempenho no programa nacional de melhoria do acesso e da qualidade da atenção básica (PMAQ), maior porcentagem de cobertura de ESF e oferta cuidado compartilhado e descentralizado na APS às PVHIV, uma referência nacional na gestão e prestação de serviços de APS^13^.

A coleta de dados ocorreu no período de julho a setembro de 2020, entrevistando inicialmente profissionais enfermeiros de quatro centros de saúde da APS e subsequentemente gestores nos setores de atenção especializada, gestão da clínica, integração assistencial e vigilância epidemiológica da Secretaria Municipal de Saúde (SMS). Conforme preconiza a TFD, optou-se pela seleção inicial de enfermeiros em razão das diferentes competências assumidas por esses profissionais no cuidado em saúde, considerados uma liderança para gestão do trabalho da equipe multidisciplinar.

Os participantes que compuseram a amostragem inicial foram selecionados intencionalmente, considerando os seguintes critérios de inclusão: atuar como enfermeiro assistencial, coordenador ou residente na APS, bem como possuir experiência na gestão do cuidado às PVHIV de no mínimo seis meses em relação à data de coleta de dados.

O convite para integrar a pesquisa foi realizado via e-mail e/ou por telefone. O termo de consentimento livre e esclarecido foi enviado virtualmente aos participantes e autorizado por meio do *Google Forms*^®^, sendo uma via arquivada com a pesquisadora.

As entrevistas intensivas aconteceram no formato não presencial devido à pandemia de covid-19, por chamada de vídeo, utilizando o sistema de comunicação *Google Meet*^®^. O primeiro grupo amostral teve como pergunta inicial: Fale-me sobre as práticas de cuidado direcionadas às PVHIV em tempos de pandemia de covid-19. Esse grupo foi composto por 12 enfermeiros.

A partir da análise das entrevistas do primeiro grupo amostral, emergiu a hipótese de que as práticas de cuidado às PVHIV durante a pandemia de covid-19 estavam relacionadas com a descentralização do manejo clínico para APS, reestruturação dos serviços e instituição de protocolos e guias de evidências científicas.

Diante disso, identificou-se a necessidade de constituir o segundo grupo amostral com gestores da SMS de Florianópolis. O objetivo das entrevistas foi explorar aspectos relacionados à instituição de protocolos de manejo clínico direcionados ao HIV, bem como mecanismos de adequação dos serviços de saúde para o cenário de pandemia de covid-19 com base no questionamento: Fale-me sobre a gestão do cuidado às PVHA em tempos de pandemia. Assim, o critério de inclusão para esse segundo grupo foi atuar em cargos de gestão na SMS de Florianópolis há mais de seis meses em relação à data de coleta de dados.

Como critério de exclusão, para os dois grupos amostrais, se considerou os profissionais que estivessem afastados do trabalho durante o período de coleta de dados, independentemente do motivo. Ao final das entrevistas do segundo grupo amostral, alcançou-se a saturação dos dados com cinco gestores e informações para sustentar o fenômeno investigado, totalizando 17 participantes.

Os dados foram coletados pelas pesquisadoras por entrevistas gravadas em áudio, que foram transcritas para o *Word*^®^ e organizadas para análise no *software NVIVO10*^®^. O tempo médio de duração de cada entrevista foi de 29 minutos. Para garantir o anonimato dos participantes, as falas foram identificadas por códigos. Para obtenção dos dados de modo a assegurar a qualidade e confiabilidade do estudo, seguiu-se os princípios do *consolidated criteria for reporting qualitative research* (COREQ).

A análise concomitante dos dados, segundo as orientações da teoria fundamentada nos dados construtivista, foi realizada em duas fases. Na primeira, foi realizada a codificação inicial, na qual os incidentes foram codificados com o intuito de compreender as informações a partir dos significados e das experiências dos participantes, constituindo as primeiras dimensões da experiência analisada. Na segunda fase ocorreu a codificação focalizada, na qual os códigos de maior expressividade foram agrupados, de modo a formar categorias abstratas para sintetizar determinado fragmento de dados^12^. Foram elaborados memorandos e diagramas para auxiliar o desenvolvimento analítico dos dados, levando ao desvelamento do fenômeno.

Esta pesquisa respeitou os preceitos éticos de pesquisa com seres humanos, segundo as Resoluções nº 466/2012 e suas complementares e, inclusive os novos procedimentos para a realização de pesquisas em ambiente virtual, de acordo com o Ofício Circular nº 02/2021.

## RESULTADOS

O Quadro apresenta as categorias e subcategorias que emergiram durante o processo de análise e que, inter-relacionadas, sustentam o fenômeno de estudo.

**Quadro:** Representação do fenômeno, categoria e subcategorias da pesquisa.

Desvelando as melhores práticas de gestão no cuidado às pessoas que vivem com HIV relacionadas com o cuidado descentralizado, compartilhado e baseado em evidências	
Categoria	Subcategorias
Desenvolvendo melhores práticas de cuidado às PVHIV frente à pandemia de covid-19	Priorizando o atendimento das pessoas que vivem com HIV.
	Facilitando as rotinas de exames (CD4, carga viral) e tratamento antirretroviral.
	Partilhando o cuidado das pessoas que vivem com HIV entre a equipe de saúde.
	Incentivando o distanciamento social das pessoas imunodeprimidas em tempos de pandemia.
	Fortalecendo o vínculo com as pessoas que vivem com HIV via grupos e redes sociais.
	Pactuando estratégias/iniciativas de cuidado intersetoriais.
	Acompanhando as pessoas que vivem com HIV pela planilha de vigilância em saúde.
	Identificando a diminuição de rastreamento de novos casos em tempos de pandemia.
	Aprendendo a utilizar ferramentas para o manejo clínico não presencial.
	Estabelecendo critérios de elegibilidade para consulta presencial e teleconsulta.
	Facilitando o acesso aos serviços de APS com teleconsulta e acolhimento via plataformas digitais.
	Utilizando protocolo para orientação do manejo clínico em teleconsulta.

*Desvelando as melhores práticas de gestão no cuidado às pessoas que vivem com HIV.*

Destacou-se a priorização e preocupação com a oferta de cuidados e acompanhamento das PVHIV.

*Atendimento priorizado! A gente tem uma comunicação interna* […] *que prevê o atendimento como prioridade a pacientes com doenças crônicas, infectocontagiosas e com demandas de urgência*. (G1E03)

Os serviços também adotaram a prática de enviar solicitações de exames e receituários via celular, o que facilita o acesso e a continuidade do cuidado em um cenário de orientação para o distanciamento social.

*As renovações de receitas estão sendo feitas todas por teleconsulta em WhatsApp.* (G1E03)

*Uma coisa que a gente fez é renovar receita de forma facilitada. Então, não é a cada renovação que o paciente precisa consultar. A gente tenta fazer a receita para mais tempo quando ele está estável. Às vezes a gente já vê que está no momento de fazer a carga viral ou CD4 e a gente já deixa o pedido junto* […] *e a consulta vai ser depois, quando ele tiver o resultado do exame.* (G1E04)

Com a descentralização do cuidado às PVHIV para a APS houve um consequente compartilhamento do cuidado entre a equipe de saúde, com divisão de responsabilidades e atribuições, inclusive em tempos de pandemia.

*A partir do momento que o paciente entrou em contato com a equipe, a gente segue dando esse acompanhamento, esse cuidado, que é geralmente mensal.* (G1E01)

*Essa gestão do cuidado é feita compartilhada com a equipe, nós médicos e enfermeiros, a gente trabalha muito juntos. A gente fala que tem um trabalho ombro a ombro.* (G1E04)

Os profissionais também enfatizaram a importância do distanciamento social para os pacientes imunodeprimidos, como as PVHIV.

*O que mudou é a entrada na unidade. A gente tem um processo de trabalho todo organizado para que as pessoas venham o mínimo possível na unidade.* (G1E03)

*A unidade se tornou um ambiente hostil para eles* [as PVHIV]*. Então, eles não queriam vir, e isso foi bom porque a gente também não queria que eles viessem.* (G1E04)

Também foram evidenciadas iniciativas relacionadas à ampliação do acesso e criação de vínculo com as PVHIV. Os participantes sinalizam as diferentes abordagens de comunicação, dentre elas se destacam os grupos e redes sociais.

*Até no Instagram tem grupo de PREP, de PEP, e sempre estão dando informações aos pacientes que são da nossa rede.* (G1E02)

*Tem um manual de uso das redes sociais com os pacientes e a gente recebeu o celular por equipe.* (G1E03)

Pensando na continuidade do cuidado e adesão ao tratamento das PVHIV foi instituída uma rede de apoio com Organizações não Governamentais (ONG) para que as pessoas que não conseguissem retirar o TARV na Central de Dispensação pudessem recebê-los em casa ou no Centro de Saúde mais próximo. Foi divulgado o telefone de quatro Centros de Testagem e Resposta Rápida (CTRR) para que os usuários pudessem comunicar suas dificuldades em relação ao tratamento e solicitar ajuda a essa rede de apoio.

*Em toda a rede tem a articulação com o Gapa*^a^ *que faz o recebimento dos medicamentos para as pessoas que não podem ir buscar devido à questão de falta de deslocamento e questões financeiras.* (G2G02)

*Uma das coisas que a gente fez foi auxiliar na retirada da TARV, que ficou impossível para algumas pessoas quando não tinha transporte coletivo. Então, eu peguei três lideranças de ONG, fiz uma reunião com eles e falei “pessoal vocês vão ter que ajudar a captar os nomes e a gente ajuda na distribuição”.* (G2G01)

*A gente divulgou muito por meio do Conselho local de saúde, dos líderes comunitários, Associação de Moradores.* (G1E04)

O monitoramento das PVHIV foi facilitado pelo uso de instrumentos de gestão, como planilhas de vigilância. Esse instrumento recebeu destaque ao possibilitar a visualização de todas as PVHIV no território, bem como sua situação de adesão ao tratamento, medicação, realização de exames, consultas, dentre outros.

*A gente está muito mais de olho na planilha de vigilância, porque ali que a gente vai acompanhar esses pacientes.* (G1E04)

*Na planilha de monitoramento tem o nome, data de nascimento, data do diagnóstico, data de quando começou a utilizar antirretrovirais, data do último CD4 e resultado, data da última carga viral e resultado.* (G1E10)

Com a priorização dos atendimentos presenciais para manejo de sintomáticos respiratórios, os profissionais identificaram uma diminuição do rastreamento de novos casos de HIV, o que é resultante da diminuição das consultas de rotina para a população sem agravos, que não foram priorizadas devido à escassez de recursos humanos.

*O que tem acontecido é que os nossos rastreamentos têm diminuído nesse momento. Então, eu tenho um paciente que não tem sintoma e que me procura para fazer o check-up: caso ele não tenha nenhuma situação de risco, ele está sendo orientado a aguardar um pouco mais. Eu tenho analisado que os nossos diagnósticos de HIV na pandemia tem sido bem poucos. Se a gente não faz sorologia, a gente vai deixar de fazer diagnóstico e vai deixar as pessoas com HIV sem saber que tem.* (G1E04)

Ainda, foram incorporadas novas maneiras de acesso à população, como o acolhimento de demandas via *WhatsApp*^Ⓡ^ , e-mail, ligação telefônica e chamada de vídeo. Desse modo, os profissionais precisaram se apoderar das maneiras de uso e funcionalidades dessas tecnologias e se adaptar ao novo modelo de cuidado imposto pela pandemia.

*É um desafio fazer* [teleconsulta] *e ter gente habilitada para fazer isso, porque não é só ter gente, às vezes eu tenho uma pessoa, mas ela não sabe escrever direito, tem dificuldade de entender.* (G1E03)

Com isso, foi instituída a prática da teleconsulta para os cuidados de rotina sem gravidade e que em primeiro momento não necessitavam de exame físico. Em situações que se necessitava um acompanhamento presencial, os usuários eram orientados a comparecerem no centro de saúde para um atendimento agendado.

*A gente faz teleconsulta via WhatsApp, tanto por vídeo, áudio, mensagem, né? Em cada caso a gente avalia a melhor forma. E isso facilita muito porque as pessoas têm que evitar sair de casa. Às vezes, são questões pontuais de pacientes que a gente já acompanha. Então, têm como dar esse suporte via teleconsulta.* (G1E01)

*Se há necessidade a gente pesa risco-benefício e faz avaliação presencial do paciente.* (G1E07)

As diversas tecnologias incorporadas no processo de trabalho em saúde melhoraram o contato com as PVHIV de modo a ampliar seu acesso aos serviços, que anteriormente ocorriam na modalidade presencial. O uso de meios digitais para a realização de teleconsulta se mostra uma ferramenta promissora para os serviços de saúde.

*Eu acho que essas novas tecnologias vêm para agregar e facilitar o acesso, muitas vezes isso facilita e melhora o vínculo em alguns casos.* (G1E08)

*A gente tem oferecido a teleconsulta como maneira de manutenção do acompanhamento no contexto da pandemia. Então, a teleconsulta é uma ferramenta que veio para somar nesse momento em que a nossa orientação é que a pessoa ficasse em casa.* (G1E04)

Foi elaborado um protocolo que autoriza e normatiza a teleconsulta como forma de colaborar no atendimento aos usuários durante a pandemia de covid-19.

*Criaram o protocolo que fala quais procedimentos éticos a gente deve realizar, como realizar uma evolução padrão, todas as orientações. Então, esse protocolo veio para dar apoio porque no início a gente não conhecia e não sabia como iria ser uma consulta pelo telefone.* (G1E05)

Pode-se compreender que os serviços de APS se reconfiguraram diante do cenário de pandemia, de modo a transformar o fluxo de trabalho, as práticas clínicas e gerenciais e incorporar novas tecnologias no processo de cuidado, garantindo continuidade da assistência às populações prioritárias para a APS. A descentralização de cuidados somada a maior autonomia dos profissionais, representam potencialidades para o desenvolvimento de melhores práticas de saúde.

A Figura sintetiza os significados atribuídos às melhores práticas de gestão no cuidado às PVHIV, e portanto, ilustra as iniciativas instituídas no cenário do estudo.

**Figura:** Desenvolvendo melhores práticas de cuidado às PVHIV frente à pandemia de covid-19.

## DISCUSSÃO

Entende-se que as melhores práticas de cuidado às PVHIV identificadas neste estudo constituem “inovações no cuidado” e estão associadas a estratégias e ferramentas instituídas na APS de Florianópolis, com destaque para a instituição de mecanismos de comunicação e de acesso aos serviços, protocolos e guias de orientação para o manejo clínico, formação de parcerias intersetoriais e realização de monitoramento e gestão do cuidado remoto. Essas iniciativas fomentam a coordenação do cuidado às PVHIV, e reforçam importantes discussões sobre os atributos da APS enquanto norteadores da política de saúde no Brasil.

É imperativo à sustentabilidade do modelo de cuidado do SUS a reorganização dos serviços de APS em tempos de pandemia de covid-19, buscando atender simultaneamente à demanda de sintomáticos respiratórios e às ações regulares que já eram acompanhadas nesse nível de atenção à saúde^14,15^. Desse modo, têm-se implementado fluxos de cuidado para o manejo desses diferentes públicos, com distinção entre quadros leves, graves e não sintomáticos respiratórios^4,5^.

O distanciamento social deve ser incentivado por todos os profissionais da equipe, principalmente pelos agentes comunitários de saúde, que têm uma aproximação maior com a comunidade, mobilizando lideranças locais para a ampla divulgação de informações e realização de medidas necessárias ao contexto^4^. Logo, evidencia-se o importante papel do trabalho comunitário e intersetorial no apoio logístico e operacional de cuidado às PVHIV em tempos de pandemia.

No cenário do estudo, a realização de parcerias comunitárias e com ONG, como o Gapa, visou o transporte e distribuição de TARV, o que resultou no acesso ao tratamento de inúmeras pessoas que se encontraram impossibilitadas de retirá-lo. Estudos apontam resultados positivos dos trabalhos desenvolvidos por organizações não governamentais destinadas à prevenção, tratamento e acompanhamento das PVHIV, na qual se destacam melhorias na redução de danos, acesso a preservativos e à TARV, vinculação ao cuidado e acesso aos serviços de saúde^16,17^. A articulação de soluções frente a cenários adversos exige uma ação coordenada no território com as lideranças, equipamentos e instituições locais, destacando-se o engajamento comunitário e as ações intersetoriais como importantes estratégias de enfrentamento da sindemia de covid-19^18^.

O conceito de sindemia consiste na interação entre duas ou mais epidemias, que produzem um aumento da carga de doenças da população^19^. Ou seja, a pandemia de covid-19 junta-se à epidemia de HIV e cria outro problema de saúde para as PVHIV, como a diminuição do acesso aos serviços de saúde. Nesse caso, onde se orienta o distanciamento social e diminuição do fluxo de pessoas nos serviços de saúde, aponta-se como estratégia a gestão do cuidado não presencial, que pode ser realizada com planilhas contendo dados clínicos e compartilhadas entre a equipe de saúde, bem como o uso de ferramentas digitais de comunicação.

O uso de instrumentos de gestão se mostra oportuno para a manutenção da adesão ao tratamento, identificação de *gaps* e acompanhamento de exames de carga viral, CD4 e CD8. Contudo, destacam-se algumas dificuldades enfrentadas para o monitoramento clínico não presencial das PVHIV, como a desatualização das informações, déficit de recursos humanos e materiais, pouca comunicação entre os profissionais da equipe, opiniões divergentes quanto à importância do acompanhamento clínico e resistências às mudanças nos processos de trabalho^20^.

A pandemia de covid-19 fez emergir tecnologias de cuidado não presencial em saúde, como a teleconsulta, que viabiliza a oferta de cuidados pelo uso das tecnologias de informação e comunicação na área saúde^21^. A implementação da teleconsulta na APS em Florianópolis permitiu garantir acesso aos usuários que necessitavam dos serviços de saúde. Desse modo, pode ser considerada uma importante ferramenta de organização assistencial para o enfrentamento da pandemia.

Ressalta-se que a instituição da teleconsulta na APS deve ser realizada com base em protocolos que regulamentam o fluxo de trabalho e estabelecem diretrizes^23^, o que requer recursos relacionados à telefonia e internet, bem como qualificação dos profissionais e usuários para usufruir dessa nova possibilidade de cuidado^22^.

O uso de aplicações como *WhatsApp*^®^ e chamada telefônica garantem a oferta de ações de forma segura^4^ favorecendo a continuidade do cuidado. Nos serviços de APS em estudo, essas tecnologias foram utilizadas em diferentes situações da atenção às PVHIV, como para responder demandas relacionadas à TARV, solicitação de exames de rotina e acolhimento de queixas clínicas.

A partir dos resultados do estudo, observa-se que a gestão do cuidado e o manejo clínico, possibilitados pelos fluxos, protocolos e pela política integrada de saúde, são fundamentais para o desenvolvimento das melhores práticas. Por meio da reestruturação da RAS, da autonomia, competência e habilidades dos profissionais e gestores nesse cenário de atuação, pode-se alcançar efetiva intervenção em fatores determinantes para o manejo das condições de saúde em tempos de covid-19.

Evidencia-se a necessidade de esforços e iniciativas para constituir uma APS forte, vigilante, capilarizada, adaptada ao contexto pandêmico e fiel aos seus princípios. A crise de saúde emergente com a pandemia de covid-19 é sanitária, política, econômica e social, bem como exige a transformação dos modos de fazer saúde^4^, sem esquecer da continuidade do acompanhamento de grupos prioritários.

Esta pesquisa analisou os significados acerca das melhores práticas de gestão no cuidado atribuídos pelos enfermeiros dos centros de saúde e gestores municipais, desse modo, apresenta limitações relacionadas a não ter sido explorada a perspectiva das PVHIV sobre o mesmo fenômeno. Também apresenta limitações quanto à realização de entrevistas online, pois se acredita que entrevistas presenciais poderiam resultar em maior interação entre entrevistador e participante, de modo a favorecer o aprofundamento do fenômeno.

Esta pesquisa contribui para o conhecimento científico sobre a gestão do cuidado no enfrentamento à pandemia de covid-19 e manutenção dos cuidados às PVHIV, especialmente no que tange às ferramentas, estratégias e tecnologias utilizadas de modo a disseminar as iniciativas implementadas na rede de saúde estudada, que têm potencial de replicação em outros cenários em que ocorrem práticas de atenção primária à saúde.

## CONCLUSÃO

Este estudo compreendeu as práticas de gestão no cuidado às PVHIV frente à pandemia de covid-19 na APS de uma capital do país, que reestruturou sua rede de serviços de saúde e implementou mudanças nas práticas clínicas e gerenciais, com o intuito de atender às novas demandas resultantes da pandemia da covid-19 e, ao mesmo tempo, manter o cuidado às PVHIV com qualidade e efetividade. Destacaram-se como melhores práticas a incorporação de tecnologias e ferramentas de gestão do cuidado não presencial como estratégia para ampliação do acesso, garantia de equidade e integralidade aos atendimentos dos usuários em um cenário no qual se orienta o distanciamento social.

Destaca-se a implantação de protocolos e fluxos para orientar o acesso aos serviços de saúde com o intuito de sistematizar as novas maneiras de prestar cuidado em tempos de pandemia, com ferramentas de natureza digital e tecnológica. A respeito disso, a utilização do *WhatsApp*^®^ ganhou destaque enquanto ferramenta para o acolhimento de demandas, realização de teleconsultas, solicitação de exames, renovação de receitas e agendamento de atividades presenciais quando necessário.

Acrescenta-se a importância da facilitação das rotinas de realização de exames de acompanhamento e de retirada da TARV. No entanto, evidencia-se que as restrições de acesso decorrentes da pandemia de covid-19 para acompanhamentos de rotina de usuários sem condições agravadas ou prioritárias, também culminaram com a diminuição do rastreamento de novos casos de HIV e controle das demais condições crônicas.

## Footnotes

a Sigla para Grupo de Apoio à Prevenção da Aids.
